# Improved access and visibility during stapling of the ultra-low rectum: a comparative human cadaver study between two curved staplers

**DOI:** 10.1186/1750-1164-6-11

**Published:** 2012-11-13

**Authors:** David E Rivadeneira, Juan Carlos Verdeja, Toyooki Sonoda

**Affiliations:** 1 Saint Catherine of Siena Medical Center, Smithtown, NY, USA; 2 Baptist Hospital of Miami, Miami, FL, USA; 3 Weill Medical College of Cornell University, New York, NY, USA

## Abstract

**Background:**

The purpose of this study was to compare in human cadavers the applicability of a commonly used stapling device, the CONTOUR® curved cutter (CC) (Ethicon Endo-Surgery, Cincinnati, OH) to a newly released, curved stapler, the Endo GIA™ Radial Reload with Tri-Staple™ Technology (RR) (Covidien, New Haven, CT)

**Methods:**

Four experienced surgeons performed deep pelvic dissection with total mesorectal excision (TME) of the rectum in twelve randomized male cadavers. Both stapling devices were applied to the ultra-low rectum in coronal and sagittal configurations. Extensive measurements were recorded of anatomic landmarks for each cadaver pelvis along with various aspects of access, visibility, and ease of placement for each device.

**Results:**

The RR reached significantly lower into the pelvis in both the coronal and sagittal positions compared to the CC. The median distance from the pelvic floor was 1.0 cm compared to 2.0 cm in the coronal position, and 1.0 cm versus 3.3 cm placed sagitally, p < 0.0001. Surgeons gave a higher visibility rating with less visual impediment in the sagittal plane using the RR Stapler. Impediment of visibility occurred in only 10% (5/48) of RR applications in the coronal position, compared to a rate of 48% (23/48) using the CC, p = 0.0002.

**Conclusions:**

The RR device performed significantly better when compared to the CC stapler in regards to placing the stapler further into the deep pelvis and closer to the pelvic floor, while causing less obstructing of visualization.

## Background

Performing surgery in the deep pelvis is often a challenging endeavor for surgeons, particularly with mobilization and division of the ultra-low/distal rectum. It is well-established that patients with distal rectal cancers have the best results with a sound oncologic approach such as the total mesorectal excision (TME) with clear circumferential and distal rectal margins. The ability to achieve clear or negative margins is of paramount importance and impacts cancer recurrence and overall patient outcomes [1-5]. Surgeons are particularly aware of the importance of proper distal rectal resection and the need to achieve with clear margins, as this may lead to a decrease in local recurrence and may increase the rate of sphincter salvage in patients with low rectal cancers [1-5]. However, the ability to place current stapling devices onto the distal rectum remains one of the most difficult challenges in pelvic surgery. This is often due to anatomical constraints such as the rigid bony confines of the pelvis and adjacent structures such as the bladder, prostate, uterus, and vagina [6-9].

Current stapling devices are fraught with a thick and bulky profile and can obscure the surgeon’s view causing difficulty in placement into the low pelvis. Improvements in stapling devices which include less bulky and lower profile designs would allow for easier placement deeper in the pelvis and onto the ultra-low rectum. This may allow for improved visualization and potentially increased distal negative margins.

In this human cadaver study we compared a commonly used stapling device, the CONTOUR® (CC) curved cutter (Ethicon Endo-Surgery, Cincinnati, OH) to the newly released Endo GIA™ Radial Reload with Tri-Staple™ Technology (RR) (Covidien, New Haven, CT) stapler. In addition, we provide significant anatomical information in regards to pelvic anatomy which may be useful for future surgical innovation.

## Methods

### Product description

#### Endo GIA™ Radial Reload with Tri-Staple™ Technology 

The RR is a curved low profile stapling device specifically designed to reach the ultra-low rectum. This disposable single patient-use stapler places two, triple staggered rows of titanium staples and simultaneously cuts the tissue creating a 60 mm curved transection.

#### CONTOUR® curved cutter

The CC stapler is a multi-fire, single patient use device with a curved head that cuts and staples. The device delivers four staggered rows of titanium staples, with a knife between the second and third row of staples, and creates a 40 mm curved transection.

### Experimental design

Twelve male cadavers were well-matched in respect to size and average weight (75 kg), and underwent a lower midline incision. The rectum was mobilized circumferentially with sharp dissection in a total mesorectal excision (TME) approach down to the pelvic floor muscles.

**Figure 1:**
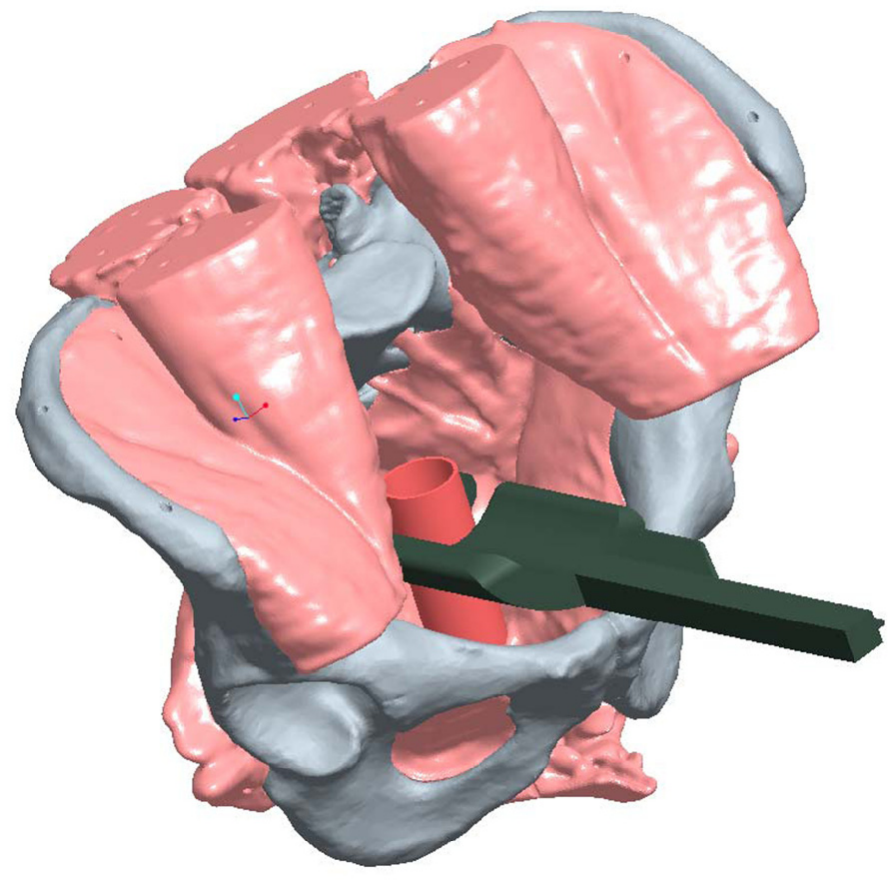
Illustration of coronal placement of CC.

**Figure 2:**
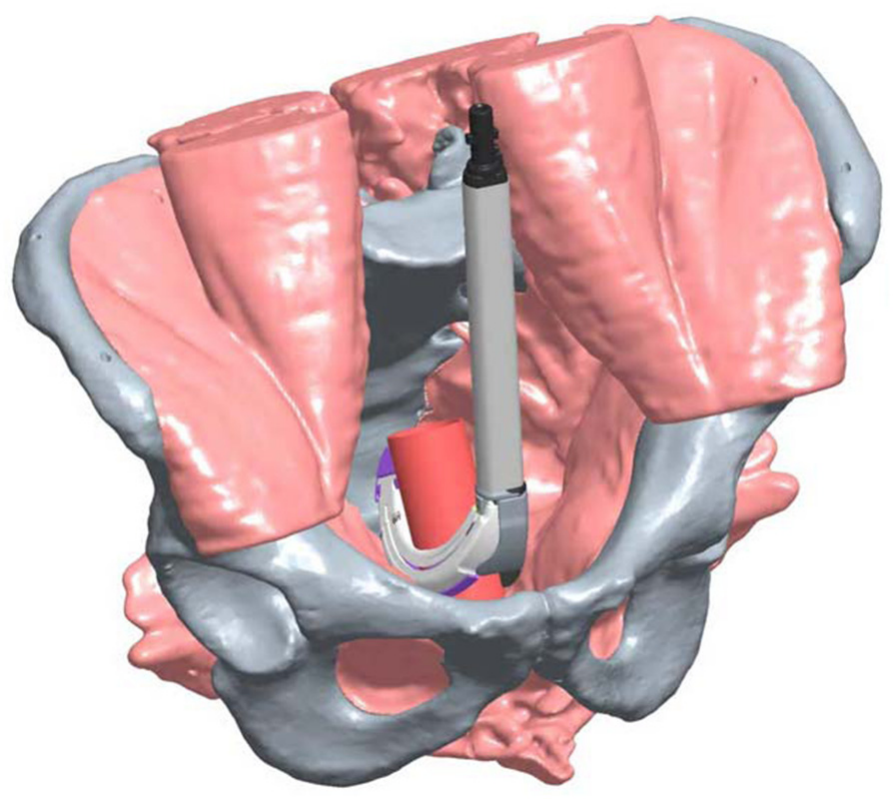
Illustration of sagittal placement of RR.

Four surgeons with extensive experience in pelvic/rectal surgery were selected to conduct the protocol procedures on each cadaver (N = 12) for a total of 48 data points. There were 12 RR staplers which were matched with 12 CC staplers. The stapling devices and orientation of placement were randomized for each of the four surgeons in order to increase study objectivity. The TME dissection proceeded to the most distal aspect of the rectum down to the pelvic floor muscles at which time both the RR and CC stapling devices were placed in the coronal and sagittal orientations **(see** Figures 1 and 2 for computer-aided drawings of stapler positioning).

The investigators recorded the distance of the stapling device from the pelvic floor. Additional data was obtained for access and visibility of the device in the sagittal and coronal position including: 1. ease of placement in the pelvis; 2. ease of placement on the colon and rectum; 3. best placement of the device (either sagittal or coronal placement); 4. ability to retain tissue 5. interference of the pubic symphysis and 6. visibility.

Extensive measurements of anatomical landmarks for each cadaver pelvis were also recorded. Distances were measured and recorded between: 1. the symphysis pubis and umbilicus, 2. the right and left anterior superior iliac spines, 3. the symphysis pubis and the sacral promontory, 4. the pelvic floor and the sacral promontory, 5. the tip of the coccyx and the symphysis pubis, 6. the right and left pelvic sidewalls (i.e., transverse diameter of the pelvic inlet), and 7. the anal verge and the pelvic floor.

Once all of the cadavers were assessed, the investigators independently determined which was the preferable device to use, and rated the access, visibility and ease of placement of the stapling devices on a scale of 1–10 (1 = poor, 10 = excellent).

#### Statistical methods

Regression models were used for statistical analysis. Linear regression was performed for continuous outcomes and logistic regression for binary and ordinal outcomes. A *p* value of < 0.05 was regarded as statistically significant. Statistical analysis was performed using SAS version 9.

## Results

**Figure 3:**
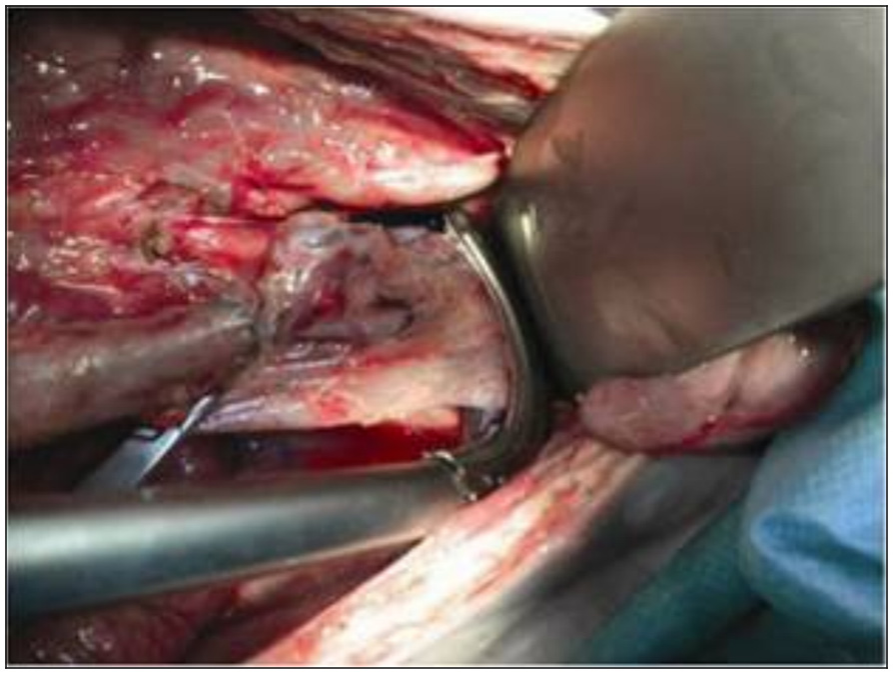
Distal Placement of RR on the Rectum.

**Figure 4:**
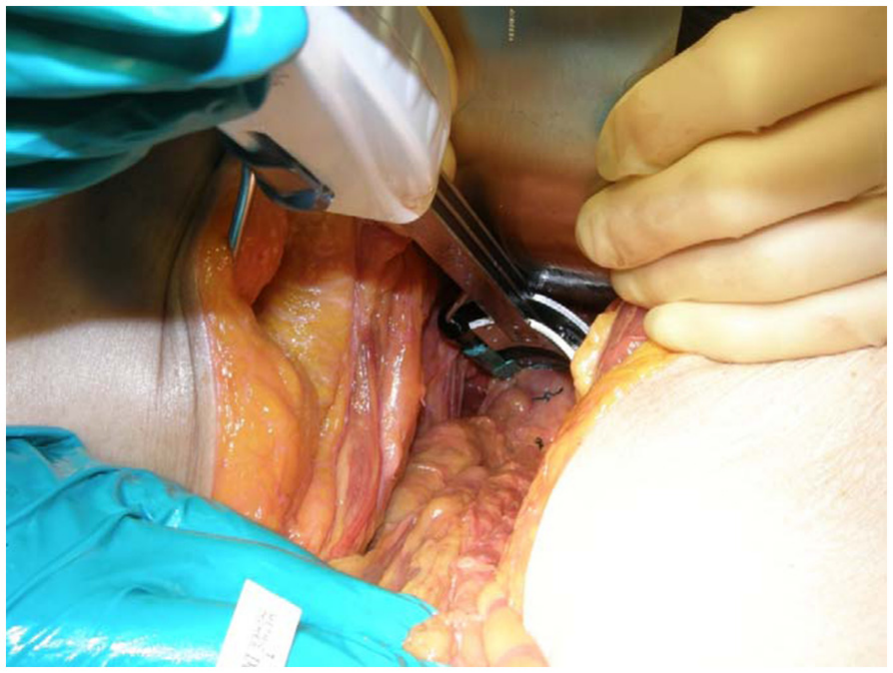
Distal Placement of CC on the Rectum.

**Figure 5:**
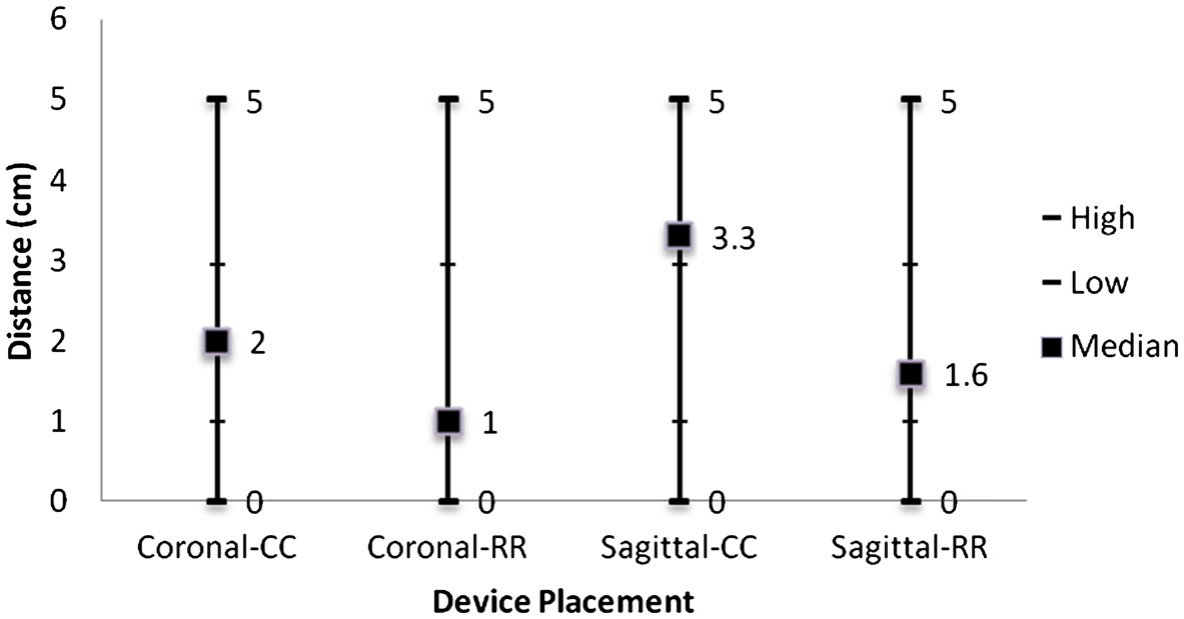
Median distance of stapler from pelvic floor.

Overall results demonstrate a statistical significance in favor of the RR stapler when compared to the CC stapler in regards to the ability to place the stapler deeper in the pelvis onto the ultra-low rectum, with an improved visibility rating in the sagittal plane and less visual impedement in the coronal plane. Surgeons were able to apply the RR lower into the pelvis in both the coronal and sagittal positions when compared to the CC stapling device (see Figures 3, 4**for RR and CC depictions, respectively)**. This was demonstrated in the coronal position with a median distance from the pelvic floor of 1.0 cm (range, 0 – 5.0 cm) for the RR , compared to 2.0 cm (0 – 5.0 cm) for the CC (p = 0.001). These significant differences for the RR stapler were also observed in the sagittal position with a median distance from the pelvic floor of 1.0 cm (0 – 5.0 cm) compared to 3.3 cm (0 – 5.0 cm) for the CC (p < 0.0001). (Figure 5)

**Figure 6:**
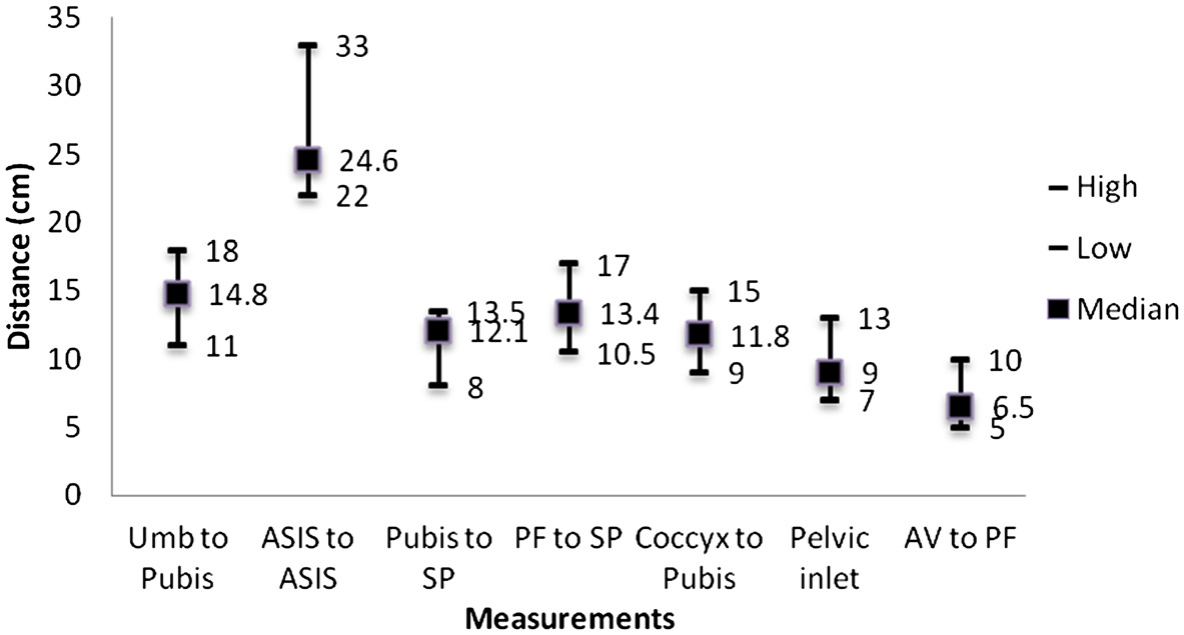
**Pelvic Measurements.** Pelvic inlet measured as transverse diameter between right and left pelvic sidewall**.** Umb = umbilicus; Pubis = pubic symphysis; ASIS = anterior superior iliac spine; SP = sacral promontory; PF = pelvic floor; Coccyx = tip of coccyx; AV = anal verge.

The RR performed significantly better than the CC when placed in both the coronal and sagittal positions with respect to the number of readjustments of the stapler that allow for optimal placement into the deep pelvis (Table 1). In addition, the RR was superior to the CC in the coronal position with respect to 1. lower incidence of interference by the symphysis pubis, 2. lower impediment of visibility, and 3. access rating. (Tables 2, 3) Applied in the sagittal position, the RR was statistically superior to the CC with respect to: 1. lower incidence of interference by the symphysis pubis, 2. ease of placement in the pelvis 3. Ability to contain the whole rectum in the device after clamping 4. visibility rating, and 5. Access rating. (Tables 4, 5) Obscured visibility was encountered 10% (5/48) of the time when the RR stapling device was used and this was favorable when compared to the higher visual impairment rate (48% [23/28]) which occurred with the CC device, p = 0.0002 (Table 2). The lack of visibility was mostly attributed to the anterior area of the deep pelvis due to the prostate in 95% of the cases. The measurements of pelvic anatomic factors in the twelve cadavers are listed in Figure 6. In regards to the ability to hold and retain tissue without slippage, the CC scored significantly higher than the RR stapler (96% [45/47] vs. 57% [26/46]).

**Table 1:** Number of readjustments for optimal placement

**Stapler placement**	**Stapler used**	**0**	**1**	**2**	**3 or more**	**P-value**
Coronal	RR	20	22	5	1	0.003
	CC	11	20	14	3	
Sagittal	RR	18	23	7	0	0.002
	CC	11	17	9	11	

**Table 2:** Yes/No Questions: Coronal placement

**Question**	**Stapler used**	**YES**	**NO**	**P Value**
Was there interference of the pubic symphysis limiting placement of the device?	RR	2 (4%)	46	0.007
	CC	13 (27%)	35	
Did the instrument impede visibility?	RR	5 (10%)	43	0.0002
	CC	23 (48%)	25	
Was the whole rectum contained in the device after clamping?	RR	46 (96%)	2	NS
	CC	46 (96%)	2	

**Table 3:** Rating questions: coronal placement

**Rating**	**Stapler used**	**Excellent**	**Adequate**	**Poor**	**P Value**
Ease of placement in the pelvis	RR	34 (71%)	8 (17%)	6 (13%)	NS
	CC	21 (44%)	26 (54%)	0	
Ease of placement around the colon and rectum	RR	29 (60%)	19 (40%)	0	NS
	CC	28 (60%)	17 (36%)	2 (4%)	
Ability to hold and retain tissue without slippage	RR	26 (57%)	12 (26%)	8 (17%)	0.0002
	CC	45 (96%)	1 (2%)	1 (2%)	
Visibility	RR	37 (77%)	9 (19%)	2 (4%)	NS
	CC	30 (63%)	14 (29%)	4 (8%)	
Pelvic access	RR	33 (69%)	11 (23%)	4 (8%)	0.008
	CC	18 (38%)	28 (58%)	2 (4%)	

**Table 4:** Yes/No Questions: Sagittal placement

**Question**	**Stapler used**	**YES**	**NO**	**P Value**
Was there interference of the pubic symphysis limiting placement of the device?	RR	4 (8%)	44	0.0006
	CC	18 (38%)	30	
Did the instrument impede visibility?	RR	2 (4%)	46	NS
	CC	6 (48%)	42	
Was the whole rectum contained in the device after clamping?	RR	43 (90%)	5	0.02
	CC	34 (70.1%)	14	

**Table 5:** Rating questions: Sagittal placement

**Rating**	**Stapler used**	**Excellent**	**Adequate**	**Poor**	**P Value**
Ease of placement in the pelvis	RR	39 (81%)	5 (10%)	4 (8%)	< .0001
	CC	13 (27%)	21 (44%)	14 (29%)	
Ease of placement around the colon and rectum	RR	29 (60%)	19 (40%)	0	NS
	CC	27 (56%)	8 (17%)	13 (27%)	
Ability to hold and retain tissue without slippage	RR	28 (62%)	10 (22%)	7 (16%)	NS
	CC	32 (68%)	2 (4%)	13 (28%)	
Visibility	RR	38 (79%)	6 (13%)	4 (8%)	0.0003
	CC	21 (44%)	14 (29%)	13 (27%)	
Pelvic access	RR	44 (92%)	1 (2%)	3 (6%)	< .0001
	CC	15 (31%)	20 (42%)	13 (27%)	

We demonstrated that the pubic symphysis interfered with the proper coronal placement of the CC stapling device more often, than the RR, occurring 27% (13/48) of the time compared to 4% (2/48) in the RR group (p = 0.007) (Table 2). These differences were also demonstrated in the sagittal placement of the stapler as there was a 38% (18/48) interference rate in the CC stapler compared to 8% (4/48) in the RR group (p = 0.006) (Table 4).

## Discussion

Particularly challenging for surgeons is pelvic dissection in patients undergoing ultra-low distal rectal procedures. The hurdles encountered in deep pelvic dissection can often be complicated by the thickness and width of the mesorectum, girth and bulk of the tumor, the rigid confines of the bony pelvis and adjacent soft tissue organs such as the prostate, uterus, and vagina [6-9]. In addition, patients with rectal cancer undergoing TME of the ultra-low rectum mandate oncologically sound techniques, including negative radial and distal margins, which have been demonstrated to significantly impact patient outcomes^1^. The ability to apply a surgical stapling device adequately into the deep pelvis in ultra-low rectal surgery with ease of use and improved visualization could provide significant benefit during TME procedures. One of these benefits could be an increase in the rate of sphincter-salvage [5,10,11]. Indeed, even 1 cm of additional clearance in the distal rectum could have significant clinical implications [5,10,11].

Currently there are several surgical stapling devices available to surgeons that allow for stapling of the distal rectum; however, many are difficult to place into the deep pelvis due to their bulky profile and do not allow for adequate visualization. An optimal stapling device for procedures involving the ultra-low rectum should have a stream-lined profile, which allows for adequate visualization in the deep pelvis and provides maximum stapling capabilities. This was the basis of our current study.

We demonstrated that the new RR stapling device improved access and visibility in the deep pelvis when compared to the CC stapler. We attribute this improvement to the design of the RR, which includes a streamlined and curved head which is more compatible with the anatomy of the deep pelvis. Furthermore, when looking at total (RR = 525 mm, CC = 406 mm) and functional access (RR = 320 mm, CC = 216) length, the RR offers significant advantages compared to the CC. One possible disadvantage of the RR stapler demonstrated in this study was difficulty holding and retaining tissue without slippage. Additional results of this study describe several pelvic measurements which may useful for future studies evaluating pelvic surgery in a difficult pelvis.

We acknowledge there are several limitations to our study. First there could be inherent bias due to the funding of the study by only one company. This could be addressed by conducting a non-industry supported multi-center study with both staplers evaluating patient outcomes. Secondly, the cadavers selected were all of average weight; therefore the influence of a high BMI was not evaluated. Therefore we could not avoid the fact that the cadavers with a lower BMI inherently allow for easier to access the pelvis. Lastly, there were only 12 cadavers utilized for the purposes of this study. A larger sample size may have yielded more statistically sound results.

Overall our study demonstrates a significant advantage of the RR stapler in accessing and visualization of the deep pelvis in a study of male cadavers and allowing for additional ultra-low rectal transection and stapling when compared to the CC stapler. Potential future studies should include investigation of the stapling device in the female pelvis and in human clinical trials exploring sphincter salvage procedures rates.

## Conclusions

The RR device performed significantly better when compared to the CC stapler in regards to placing the stapler further into the deep pelvis and closer to the pelvic floor, while causing less obstructing of visualization. The ability to apply a surgical stapling device further distally in the pelvis onto the ultra-low rectum with improved visibility in the sagittal plane and less visual impediment in the coronal plane may potentially aid in achieving clear or negative distal margins. Proper distal rectal resection and the need to achieve clear margins may lead to a decrease in local recurrence and may increase the rate of sphincter salvage in patients with low rectal cancers.

## Competing interests

David E. Rivadeneira, MD: Received compensation as a consultant and has a consultant agreement with Covidien. Received honoraria from Covidien for speaking events, laparoscopic educational courses, and hourly compensation for work on this cadaver project and manuscript preparation. There is no ownership of stocks, stock options, or equity interests, patent- licensing agreements, or research support. Currently a consultant with Ethicon as well.

Juan-Carlos Verdeja, MD: No financial interests, consultant agreements, or speaker bureau agreements with Covidien and Ethicon. Received hourly compensation from Covidien for work on this cadaver project.

Toyooki Sonoda, MD: Received honoraria from Covidien for speaking events, lectures at laparoscopic educational courses, and hourly compensation for work on this cadaver project. There is no consultation agreement, stock or stock option ownership, patent-licensing agreements, or research support. There is no financial association with Ethicon.

As a group of four participating surgeons, hourly compensation for work on the cadaver laboratory and manuscript preparation totaled $22,950.

## Acknowledgements

The authors acknowledge Jessica Chowaniec, clinical research associate at Covidien, who was indispensible in the conception, design and acquisition of data. The authors thank Ping-Yu Liu, Ph.D for statistical analysis. The acquisition of cadavers, surgical instruments, and stapling instruments were funded by Covidien. All authors were reimbursed by Covidien for time spent conducting the research project, and for preparation and review of the manuscript.

## Authors' contributions

DR was involved in the study design, data acquisition, analysis, interpretation of data, and drafting of the manuscript. JV and TS were involved in the study design, data acquisition, analysis, interpretation of data, and critical revision of the manuscript. All authors read and approved the final manuscript.
